# Proteomic Analysis Reveals a Biofilm-Like Behavior of Planktonic Aggregates of *Staphylococcus epidermidis* Grown Under Environmental Pressure/Stress

**DOI:** 10.3389/fmicb.2019.01909

**Published:** 2019-09-06

**Authors:** Marta Bottagisio, Alessio Soggiu, Cristian Piras, Alessandro Bidossi, Viviana Greco, Luisa Pieroni, Luigi Bonizzi, Paola Roncada, Arianna B. Lovati

**Affiliations:** ^1^IRCCS Istituto Ortopedico Galeazzi, Laboratory of Clinical Chemistry and Microbiology, Milan, Italy; ^2^Department of Veterinary Medicine (DiMeVet), University of Milan, Milan, Italy; ^3^Institute of Biochemistry and Clinical Biochemistry, Università Cattolica del Sacro Cuore Roma, Rome, Italy; ^4^Fondazione Policlinico Universitario Agostino Gemelli IRCCS, Rome, Italy; ^5^Proteomics and Metabonomics Unit, IRCCS Fondazione Santa Lucia, Rome, Italy; ^6^Department of Health Sciences, Università degli Studi “Magna Græcia”, Catanzaro, Italy; ^7^IRCCS Istituto Ortopedico Galeazzi, Cell and Tissue Engineering Laboratory, Milan, Italy

**Keywords:** proteomics, methicillin-resistant *Staphylococcus epidermidis*, biofilm, planktonic, sessile, prosthetic joint infections, orthopedics

## Abstract

Prosthetic joint replacement failure has a huge impact on quality of life and hospitalization costs. A leading cause of prosthetic joint infection is bacteria-forming biofilm on the surface of orthopedic devices. *Staphylococcus epidermidis* is an emergent, low-virulence pathogen implicated in chronic infections, barely indistinguishable from aseptic loosening when embedded in a mature matrix. The literature on the behavior of quiescent *S. epidermidis* in mature biofilms is scarce. To fill this gap, we performed comparative analysis of the whole proteomic profiles of two methicillin-resistant *S. epidermidis* strains growing in planktonic and in sessile form to investigate the molecular mechanisms underlying biofilm stability. After 72-h culture of biofilm-forming *S. epidermidis*, overexpression of proteins involved in the synthesis of nucleoside triphosphate and polysaccharides was observed, whereas planktonic bacteria expressed proteins linked to stress and anaerobic growth. Cytological analysis was performed to determine why planktonic bacteria unexpectedly expressed proteins typical of sessile culture. Images evidenced that prolonged culture under vigorous agitation can create a stressful growing environment that triggers microorganism aggregation in a biofilm-like matrix as a mechanism to survive harsh conditions. The choice of a unique late time point provided an important clue for future investigations into the biofilm-like behavior of planktonic cells. Our preliminary results may inform comparative proteomic strategies in the study of mature bacterial biofilm. Finally, there is an increasing number of studies on the aggregation of free-floating bacteria embedded in an extracellular matrix, prompting the need to gain further insight into this mode of bacterial growth.

## Introduction

Prosthetic joint replacement is one of the most widely performed orthopedic procedures and offers effective therapeutic options in the treatment of severe osteoarthritis. Today, arthroplasty enjoys high success rates and provides long-term pain relief and restoration of knee or hip joint function (Cooper, 2014). Despite the excellent clinical results, prosthetic joint replacements are notoriously burdened by complications, including persistent pain, implant loosening, and infection, ultimately requiring revision surgery. Prosthetic joint infections (PJIs) are one of the major causes of implant failure. Implant replacement affects quality of life and hospitalization costs (Drago et al., 2012).

An aging population means a rise in total hip and knee arthroplasties and the number of PJI cases. It has been estimated, in fact, that a PJI develops in 1–2% of primary arthroplasties and 5–40% of revision surgeries (Trampuz and Widmer, 2006). PJIs usually derive from accidental contamination in the operating room, and the causative microorganisms that colonize the implant and form biofilm on its surface are primarily *Staphylococcus aureus*, *Staphylococcus epidermidis*, and *Pseudomonas aeruginosa* (Trampuz and Widmer, 2006). Staphylococci account for 82.3% of clinically isolated bacteria, while *S. aureus* and *S. epidermidis* infections account for 31.7 and 39% of all isolates obtained from implants, respectively (Arciola et al., 2015).

*Staphylococcus epidermidis* has recently been identified as an emergent, low-virulence pathogen implicated in nosocomial infections associated with medical devices (e.g., catheters, pacemakers, metal implants) (Ziebuhr et al., 2006). *S. epidermidis* is a commensal Gram-positive, coagulase-negative bacterium. Depending on the biological context in which it grows, it can be either a symbiont or a pathogen in chronic infection characterized by the absence of specific clinical signs and barely distinguishable from aseptic prosthetic failure (Lovati et al., 2017). Successful treatment relies on establishing whether the case is related to aseptic loosening or implant infection. Unfortunately, the diagnostic criteria for PJIs are based on tests that are not reliably predictive for implant-associated infections (e.g., C-reactive protein, erythrocyte sedimentation rate) (Berger et al., 2017), which poses diagnostic challenges especially when confronted with a chronic state not characterized by severe signs of infection caused by low-virulence bacteria like *S. epidermidis* (Drago and De Vecchi, 2017; Li et al., 2018).

Unlike *S. aureus*, *S. epidermidis* does not encode many pathogenicity islands; its major virulent property is the ability to establish organized communities that regulate the expression of genes involved in survival mechanisms such as forming biofilm on implants (Patel, 2005; Fey and Olson, 2010). Furthermore, biofilm confers *S. epidermidis* a protective niche in which sessile bacteria can grow and evade the host’s immune defenses and antimicrobial treatments, leading to the development of antimicrobial-resistant strains such as methicillin-resistant *S. epidermidis* (MRSE) (Patel, 2005; Heilmann et al., 2018). The complete pathway that regulates biofilm formation *in vivo* is subdivided into four progressive steps in the expression of specific proteins: attachment, accumulation, maturation, and detachment in which bacteria separate from the mature matrix to spread the infection (Otto, 2008; Büttner et al., 2015).

The literature is scant on the behavior of quiescent cells embedded in mature biofilms. To fill this gap, we wanted to identify the proteins expressed by a mature biofilm on metallic implants by comparing the whole proteomic profiles of two different strains of device-related MRSE grown in plankton and in sessile form. Analysis of the proteins expressed in these different culture conditions after 72 h of growth disclosed the mechanisms behind the biofilm stability and the differences between the two bacterial strains. The preliminary study results for the characterization of prokaryotic cell regulation may lead to the identification of potential diagnostic biomarkers or therapeutic targets to detect latent and chronic infections mediated by low-virulence pathogens such as *S. epidermidis*.

## Materials and Methods

### MRSE Strains, Culture Conditions, and Sampling

Two different strains of MRSE were used. The reference *S. epidermidis* strain (ATCC 35984) was obtained from the American Type Culture Collection (Manassas, VA, United States). Differently, the clinical MRSE strain (GOI1153754-03-14) was isolated at the Center for Reconstructive Surgery of Osteoarticular Infections (CRIO) and subsequently characterized at the Laboratory of Clinical Chemistry and Microbiology of the IRCCS Galeazzi Orthopedic Institute (Milan, Italy), as described elsewhere (Lovati et al., 2016; Bottagisio et al., 2017). The ability of MRSE GOI1153754-03-14 to colonize implants was recently validated in an *in vivo* study (Lovati et al., 2016); the whole genome sequence of the clinical isolate revealed that biofilm formation is regulated by the expression of polysaccharide intercellular adhesion (PIA) encoded by the icaADBC and the icaR regulatory genes (Bottagisio et al., 2017).

Both MRSE strains were cultured in their planktonic and sessile form. Briefly, 1.5 × 10^8^ CFU/ml of MRSE GOI1153754-03-14 or ATCC 35984 were grown under vigorous agitation (300 rpm) in brain heart infusion broth (BHI, bioMérieux, Marcy-l’Étoile, France) at 37°C under aerobic conditions. After 72 h, the bacterial suspension was centrifuged at 3000 rpm for 10 min at 4°C to obtain a triplicate 50 mg of bacterial pellet of planktonic cultures. The cell pellets were carefully washed six times with ice-cold PBS, the supernatant was removed, and the pellets were stored at *−*20°C until use. The sessile cultures were grown on sandblasted titanium disks to resemble the bacterial biofilm formation on prosthetic implants, as previously reported (Drago et al., 2012). Briefly, sterile sandblasted titanium disks (Ø 25 mm; thickness 5 mm) (Adler Ortho, Cormano, Italy; batch J04051) were incubated in six-well plates containing 5 ml of fresh BHI and approximately 1.5 × 10^8^ CFU/ml of MRSE GOI1153754-03-14 or ATCC 35984. The plates were statically incubated at 37°C under aerobic conditions for 72 h, the titanium disks were then washed three times with ice-cold PBS to remove any floating bacteria and scraped with a sterile silicone cell scraper (VWR International, Milan, Italy) on ice. The bacterial suspension was centrifuged and washed to obtain a triplicate of 50 mg of bacterial pellet. All the samples were stored at −20°C until analysis.

### Protein Extraction and Quantification

The bacterial pellets obtained from the planktonic and sessile cultures were suspended at a ratio of 1:10 (w/v) in rehydration buffer containing 7 M urea, 2 M thiourea, and 2% 3-[(3-cholamidopropyl) dimethylammonio]-1-propanesulfonate hydrate (CHAPS) supplemented with a mix of protease inhibitors and nucleases (GE Healthcare, Little Chalfont, Buckinghamshire, United Kingdom) according to the manufacturer’s instructions. The samples were processed with six cycles of 60-s bead beating at 4,000 rpm (MiniLys, Bertin Technologies; Montigny-le-Bretonneux, France) using 0.1-mm zirconium silica beads (BioSpec, Bartlesville, OK, United States), added in a ratio of 1:1 (w/v) to the pellet suspension, interspersed by 5 min cooling on ice and 5 min centrifugation at 2°C and 20,000 *g*. After the bead beating cycles, the samples were centrifuged at 20,000 *g* at 2°C for 30 min. The supernatants were collected and the protein concentration in the samples was determined using Bradford assay (Bio-Rad protein assay, Bio-Rad, Hercules, CA, United States). Absorbance was measured using a spectrophotometer (Gene Quant 100, GE Healthcare) at 595 nm. The extracted proteins were stored at −80°C until use.

### Two-Dimensional Electrophoresis (2-DE)

Proteins were separated using two-dimensional electrophoresis (2-DE). For the isoelectric focusing (IEF) step, immobilized pH gradient (IPG) polyacrylamide gel strips (GE Healthcare, 7 cm, pH 4.0–7.0) and Protean IEF Cell (Bio-Rad) were used. Prior to IEF, 100 μg of protein sample was dissolved in a solution containing 7 M urea, 2 M thiourea, 2% w/v CHAPS, 30 mM DTT, 0.5% w/v ampholine (pH 3.5–10.0), and 1% w/v bromophenol blue. The IPG strips were actively rehydrated with the sample at 50 V and 20°C for 16 h. After rehydration, paper wicks soaked in milliQ water (8 μl) were placed between the cathode, the anode, and the gel strip to prevent the strips from burning due to the high voltage. The voltage was gradually increased as follows: 100 V (4 h), 250 V (2 h), 5000 V (5 h), and 5000 V until the cumulative voltage reached 50 kVh. A limit of current up to 50 μA per gel strip was set. Following IEF, each strip was reduced for 15 min in 5 ml of solution containing 6 M urea, 2% w/v SDS, 50 mM Tris–HCl buffer, pH 8.8, and 30% v/v glycerol with 1% w/v DTT added, and then alkylated in 5 ml of the same solution with 2.5% w/v of IAA added in place of DTT. The IPG strips were then washed quickly in 1 × running buffer (25 mM Tris–HCl, pH 8.8, 192 mM glycine, 1% w/v SDS, and milliQ water), loaded onto 10% w/v polyacrylamide-resolving gels along with the protein ladder and fixed with 0.5% w/v low-melting-point agarose gel. The second dimension was carried out in Mini-PROTEAN^®^ Tetra cell system (Bio-Rad). In the first step of electrophoresis, 8 mA per gel were applied for 15 min until the bromophenol blue front line entered the resolving gel. In the second step, 16 mA per gel were applied until the bromophenol blue front line reached the bottom of the gel. The gels were stained overnight in 100 ml of Coomassie Blue G-250 (Sigma-Aldrich, St. Louis, MO, United States).

### Image Acquisition and Analysis

A series of 2-DE maps were acquired using a flatbed densitometer (ImageScanner III, GE Healthcare, Uppsala, Sweden). Variations in protein expression were analyzed using Progenesis SameSpots Version 4.6 software (Non-linear Dynamics, Newcastle upon Tyne, United Kingdom). The module for 2-DE gel analysis was used for image aligning, background removal and detection, normalization, and matching of the spots.

### Protein Identification

Protein identification was carried out as previously described (Piras et al., 2015). Briefly, analysis was performed on an Ultraflex III MALDI-TOF/TOF spectrometer (Bruker-Daltonics; Billerica, MA, United States) in positive reflectron mode. For external calibration, the standard peptide mixture calibration (Bruker-Daltonics: m/z: 1,046.5418, 1,296.6848, 1,347.7354, 1,619.8223, 2,093.0862, 2,465.1983, 3,147.4710) was used. To select monoisotopic peptide masses, mass spectra were analyzed with FlexAnalysis 3.3 software (Bruker-Daltonics). After internal calibration (known autolysis peaks of trypsin, m/z: 842.509 and 2,211.104) and exclusion of contaminant ions (known matrix and human keratin peaks), the peak lists were analyzed by MASCOT version 2.4.1 algorithm^1^ against Uniprot/SwissProt database 2018_11 restricted to *S. epidermidis* reviewed taxonomy (2,539 sequences). For the database search, the parameters carbamidomethylation of cysteines and oxidation on methionines were set for the fixed and variable modifications, respectively; one missed cleavage site was set for trypsin, and maximal tolerance was established at 70 ppm. For protein identification assignment, only Mascot scores >56 were considered significant (*p* < 0.05). To confirm the identification obtained, MS/MS spectra were acquired by switching the instrument in LIFT mode with 4–8 × 10^3^ laser shots using the instrument calibration file. For fragmentation, the precursor ions were manually selected and the precursor mass window was automatically set. For each MS/MS spectra acquired, spectra baseline subtraction, smoothing (Savitzky–Golay), and centroiding were operated using Flex-Analysis 3.3 software. The following parameters were used for the database search: carbamidomethylation of cysteines and oxidation on methionine were set for fixed and variable modifications, respectively, maximum of one missed cleavage was established, and the mass tolerance was set to 50 ppm for precursor ions and to a maximum of 0.4 Da for fragments. The confidence interval for protein identification was set to 95% (*p* < 0.05), and only peptides with an individual ion score above the identity threshold were considered correctly identified.

### Liquid Chromatography High-Definition Mass Spectrometry^E^ (LC-HDMSE) Analysis

Protein digestion was performed according to the filter-aided sample preparation (FASP) protocol (Wiśniewski et al., 2009; Distler et al., 2016) that combines both protein purification and digestion. Each biological sample was run in quadruplicate. Briefly, reduction (DTT 8 mM in urea buffer-8 M urea and 100 mM Tris), alkylation (IAA 50 mM in urea buffer-8 M urea and 100 mM Tris), and digestion by trypsin at a final concentration of 0.01 μg/μl (Promega Italia srl, Milan, Italy) were performed on filter tubes (Nanosep centrifugal device with Omega membrane-30 K MWCO, Sigma-Aldrich). LC-MS analysis was performed as previously described (Greco et al., 2018). First, 500 fmol/μl of digestion of enolase from *Saccharomyces cerevisiae* (P00924) was added to each sample as an internal standard, tryptic peptides were separated, and then 0.25 μg of each digested sample was loaded onto a Symmetry C18 5 μm, 180 μm × 20 mm precolumn (Waters Corp., Milford, MA, United States) and subsequently separated by a 90-min reversed-phase gradient at 300 nl/min (linear gradient, 2–85% CH_3_CN over 90 min) using a HSS T3 C18 1.8 μm, 75 μm × 150 mm nanoscale LC column (Waters Corp.) maintained at 40°C. The separated peptides were analyzed on a high-definition Synapt G2-Si Mass spectrometer directly coupled to the chromatographic system. Protein expression was evaluated via a label-free ion mobility–enhanced data-independent acquisition (DIA) proteomics analysis in expression configuration mode (HDMS^E^). Processing of low and elevated energy, added to the data of the reference lock mass [Glu1]-Fibrinopeptide B Standard (Waters Corp.), provided a time-aligned inventory of accurate mass-retention time components for both the low- and the elevated-energy exact mass retention time (EMRT).

### Label-Free Data Analysis

Label-free protein quantification was performed using Progenesis QI for Proteomics v4.0.6403.35451 (Waters Ltd., Newcastle upon Tyne). The samples were automatically aligned according to retention time. The peak processing method was performed in profile data mode and the peptide ion detection method was set in high-resolution mode. Peptides with charges between 2 + and 7 + were retained. Database search was performed using the ion accounting method against a custom-made Uniprot *S. epidermidis* RP62A reviewed database (peptide mass tolerance 10 ppm and fragment ion tolerance 0.01 Da). Carbamidomethyl cysteine and oxidation of methionine were selected as fixed and variable modifications, respectively. The search results were filtered to obtain a protein false discovery rate of 1%. Protein quantification was based on relative quantitation using the Hi-N method (*n* = 3) and averaging the individual abundances for every unique peptide for each protein and comparing the relative abundance across sample runs and between the experimental groups. Proteins were considered differentially expressed according to the following criteria: protein identified in at least three out of four runs of the same sample with a fold change of regulation >± 20%; only modulated proteins with a *p*-value <0.05 [according to analysis of variance (ANOVA)] were considered significant (Greco et al., 2018).

### Bioinformatics Analysis

The ClueGO Cytoscape plugin 2.5.4 (Bindea et al., 2009) and CluePedia 1.5.4 (Bindea et al., 2013) were used to obtain functional interaction networks starting from the statistically significant over- and underexpressed proteins in each experimental group. Functions associated with the groups were partitioned based on significant functional associations between terms and protein sets. Gene ontology (GO) categories and pathways included biological processes (BPs), molecular functions (MFs), and Kyoto Encyclopedia of Genes and Genomes (KEGG) updated at the last release. Redundant terms were grouped based on a kappa score of 0.4 (Bindea et al., 2009). The *p*-value was calculated and corrected with a Bonferroni step down. Only pathways with a *p*-value ≤0.05 were selected. These analyses were carried out based on the *S. epidermidis* RP62A annotations. Network visualization was performed on Cytoscape version 3.7.1 (Shannon et al., 2003). Venn diagrams were drawn using the Venny web service^2^.

### Microbial Cytology

Following analysis of the proteomic profile of planktonic bacteria, cytological evaluation of the behavior of *S. epidermidis* was conducted after 72 h of culture. Briefly, both MRSE GOI1153754-03-14 and ATCC 35984 were grown under vigorous agitation (200 rpm) in BHI broth (bioMérieux) at 37°C under aerobic conditions to mimic the previously described experimental design. An aliquot of bacteria was then collected at 24, 48, 72, and 96 h and the behavior of the bacteria was evaluated by cytological staining. After heat fixation, the bacteria were marked with Gram staining to assess cell morphology and arrangement and with Alcian blue staining to appreciate any possible matrix production (McKinney, 1953). Photomicrographs were acquired using an Olympus IX71 light microscope with a 100× oil immersion objective with a digital camera (Olympus, Corp. Tokyo, Japan).

### Confocal Laser Scan Microscopy Analysis

Sessile and planktonic forms of MRSE GOI1153754-03-14 and ATCC 35984 were analyzed by confocal laser scan microscopy (CLSM). Briefly, planktonic and sessile cultures were grown as described above. After 72 h of incubation, the samples were stained with Filmtracer^TM^ LIVE/DEAD^TM^ Biofilm Viability Kit (Thermo Fisher Diagnostics, Waltham, MA, United States) according to the manufacturer’s instructions. Briefly, a staining solution was prepared by adding 1 μl of SYTO9 and 3 μl of propidium iodide to 1 ml of sterile water. The planktonic samples were stained by incubating 10 μl of bacterial suspension with an equal volume of staining solution and let to dry in the dark at room temperature. Differently, the titanium discs were gently washed three times with sterile saline to remove any non-adherent cells. The samples were incubated with 200 μl of staining solution at room temperature in the dark for 15 min. After incubation, the samples were washed again with sterile saline to remove any excess dye and let to dry under a laminar flow hood. The planktonic and sessile samples were then examined with upright CLSM TCS SP8 (Leica Microsystems CMS GmbH, Mannheim, Germany). A 488-nm laser line was employed to excite SYTO9 and a 552-nm line was used to excite propidium iodide. Sequential optical sections were collected along the *z*-axis over the complete thickness of the sample. Images from at least three randomly selected areas were acquired for each disc with a 20× objective. The images were then processed with Las X software (Leica Microsystems CMS GmbH) and analyzed with Fiji software (Fiji, ImageJ, Wayne Rasband National Institutes of Health). The live/dead cell ratio was assessed as previously reported (Bidossi et al., 2017).

### Statistical Analysis

Statistical analysis for 2D gel data was performed using the Progenesis Stats module on the log-normalized volumes for all spots. The Progenesis stats module automatically performs one-way ANOVA on each spot to evaluate the *p*-value between different groups; for this study, *p*-values <0.05 were considered statistically significant. As indicated above, the differential proteomic analysis for label-free data was done by analyzing all the proteins identified in the experimental groups. All probability values were calculated using one-way ANOVA; *p*-values <0.05 were considered statistically significant.

## Results

### Proteomics

**FIGURE 1:**
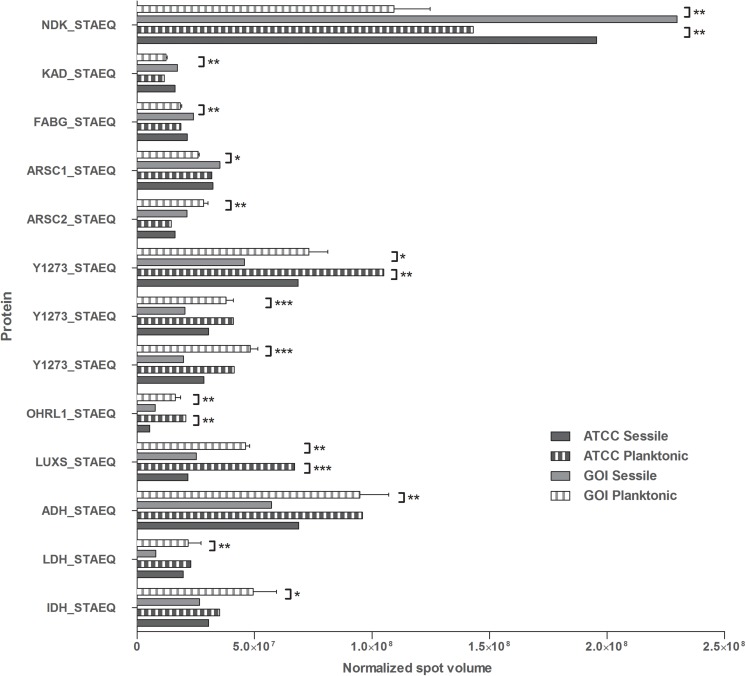
Quantification of the identified proteins. The histograms present the normalized volumes of the spots processed by Progenesis SameSpots software. Data are expressed as mean ± SD. Statistical significance for ^∗^*p* < 0.05, ^∗∗^*p* < 0.01, and ^∗∗∗^*p* < 0.001.

All 2D maps resolved approximately 573 ± 10 protein spots. Gel imaging analysis showed that 16 proteins were differently expressed in the planktonic and the sessile bacteria. Table 1 presents the strains and culture conditions, along with information on sequence coverage, Mascot score, and peptide match. Figure 1 presents quantification of the normalized spot volume.

**TABLE 1:** List of significant proteins identified in planktonic and sessile *S. epidermidis* by 2-DE and confirmed by label-free analysis.

**UniProt ID**	**UniProt accession number**	**Protein name**	**EMW/MW^a^**	**Sequence coverage^b^**	**Mascot score^c^**	**Peptide match**	**ATCC sessile vs. planktonic^d^**	**GOI sessile vs. planktonic^d^**
NDK_STAEQ	Q5HP76	Nucleoside diphosphate kinase	16.75/16.75	34	86	7/44	↑ (0.0082)	↑ (0.0082)
KAD_STAEQ	Q5HM20	Adenylate kinase	24.02/24.03	32	66	10/42	↓ (HDMS^E^)	↑ (0.0115)
FABG_STAEQ	Q5HPW0	3-oxoacyl-[acyl-carrier-protein] reductase FabG	26.07/26.07	36	86	11/44	↓ (HDMS^E^)	↑ (0.0013)
ARSC1_STAEQ	Q5HRI4	Arsenate reductase 1	14.65/14.7	32	102	6/48	–	↑ (0.0318)
ARSC2_STAEQ	Q5HKB7	Arsenate reductase 2	14.69/14.7	40	88	10/48	↓ (HDMS^E^)	↓ (0.0062)
Y1273_STAEQ	Q5HNJ5	Putative universal stress protein SERP1273	18.42/18.47	56	108	9/67	↓ (0.0085)	↓ (0.0423)
Y1273_STAEQ	Q5HNJ5	Putative universal stress protein SERP1273	18.42/18.47	58	110	10/67	↓ (HDMS^E^)	↓ (<0.001)
Y1273_STAEQ	Q5HNJ5	Putative universal stress protein SERP1273	18.42/18.47	64	120	12/67	↓ (HDMS^E^)	↓ (<0.001)
OHRL1_STAEQ	Q5HQR8	Organic hydroperoxide resistance protein-like 1	15.35/15.46	35	70	6/65	↓ (0.0102)	↓ (0.0013)
LUXS_STAEQ	Q5HM88	S-ribosylhomocysteine lyase^∗^	17.64/17.08	32	86	6/41	↓ (0.0010)	↓ (0.0091)
ADH_STAEQ	Q5HRD6	Alcohol dehydrogenase	36.45/36.83	49	86	15/67	↓ (HDMS^E^)	↓ (0.0088)
LDH_STAEQ	Q5HL31	L-lactate dehydrogenase	34.10/34.14	33	68	10/48	↓ (HDMS^E^)	↓ (0.0053)
IDH_STAEQ	Q5HNL1	Isocitrate dehydrogenase	46.62/46.64	37	120	16/74	↓ (HDMS^E^)	↓ (0.0283)

*^*a*^Estimated molecular weight/molecular weight (EMW/MW) expressed in kDa. ^*b*^Data referring to sequence coverage are expressed as percentage. ^*c*^Mascot scores were obtained against *S. epidermidis* ATCC 35984 sequence presented in the database. ^*d*^Differences in the protein expression of sessile bacteria compared to their planktonic counterparts, along with their *p* values. ^∗^LuxS was overexpressed in the clinical sessile group based on HDMS^*E*^ data.*

Four of the 13 proteins showed increased expression as the result of biofilm development; nucleoside diphosphate kinase (Ndk, NDK_STAEQ–Q5HP76) was identified in both MRSE GOI1153754-03-14 and ATCC 35984. Similarly, adenylate kinase (adk, KAD_STAEQ–Q5HM20) was also overexpressed after 72-h culture on the titanium disks. Together with the higher expression of 3-oxoacyl-[acyl-carrier-protein] reductase FabG (FABG_STAEQ–Q5HPW0), expression of these proteins suggested active metabolism of sessile bacteria involved in the synthesis of nucleoside triphosphate and polysaccharides.

Moreover, we found overexpression of arsenate reductases 1 and 2 (ArsC 1 and 2, ARSC1_STAEQ–Q5HRI4; ARSC2_STAEQ–Q5HKB7) in the sessile and the planktonic bacteria, respectively. Both ArsCs exhibit the protein tyrosine phosphatases I (PTPases I) fold typical of low-molecular-weight tyrosine phosphatases (LMW PTPases) (Zegers et al., 2001). Overall, the data revealed that most of the changes in the proteomic profile of both *S. epidermidis* strains occurred when planktonically cultured. A remarkable difference was found between the cells in response to stress: the planktonic cells expressed higher levels of putative universal stress protein (Y1273_STAEQ–Q5HNJ5) than their sessile counterpart. Only one of the three detected isoforms was shared between the two MRSE strains.

Similarly, another cytoplasmic protein expressed in response to oxidative stress, hydroperoxide resistance protein-like 1 (OHRL1_STAEQ–Q5HQR8), was underexpressed in both *S. epidermidis* strains when grown in sessile form. Once again, the expression of S-ribosylhomocysteine lyase (LuxS, LUXS_STAEQ–Q5HM88), a regulator of the quorum sensing (QS) system by planktonic bacteria, confirmed the harsh culture conditions. Not only was there a shortage of nutrient and an accumulation of cells and catabolites, there were also low oxygen levels due to the overexpression of the two enzymes alcohol dehydrogenase (Adh, ADH_STAEQ–Q5HRD6) and L-lactate dehydrogenase (Ldh, LDH_STAEQ–Q5HL31) involved in the fermentative pathway.

Finally, the presence of a considerable amount of isocitrate dehydrogenases (IDH_STAEQ–Q5HNL1) suggested physiological heterogeneity of the bacterial populations in the culture conditions. Label-free analysis confirmed the trend of all the proteins identified by 2-DE, except for LuxS, which showed overexpression in the sessile isolates (Supplementary File 1) and a non-significant trend (data not shown) to overexpression in the planktonic ATCC 35984. Furthermore, label-free analysis enabled us to retrieve and confirm the missing 2D data for the ATCC strain that were not detected in the 2-DE experiment (Table 1). The proteins missing in the 2DE experiment from the ATCC group were overexpressed in the planktonic group.

**FIGURE 2:**
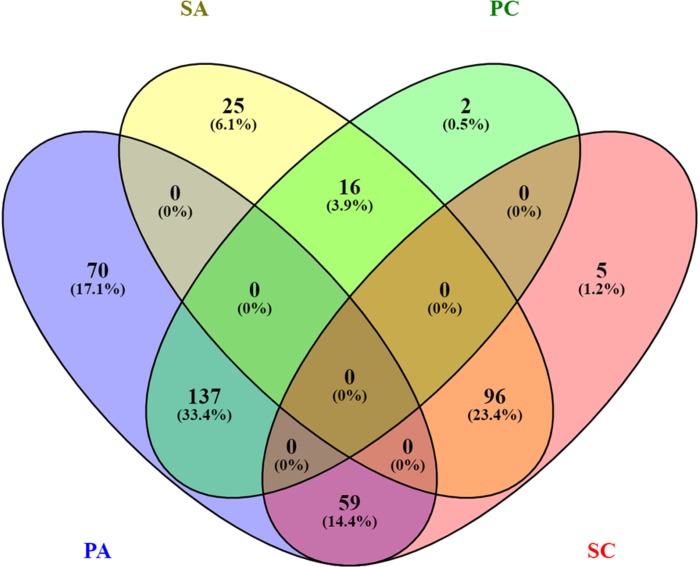
Venn diagram showing the distribution of statistically significant proteins in the four experimental conditions: planktonic ATCC (PA), planktonic clinic (PC), sessile ATCC (SA), and sessile clinic (SC), respectively.

In this study, we investigated the proteome dynamics of the planktonic (PA, PC) and sessile forms (SA, SC) of *S. epidermidis* ATCC 35984 and clinical isolates, respectively. For each condition, four biological replicates were analyzed. The proteins were extracted and digested from each experimental sample as described in Materials and Methods, and the resulting peptides were analyzed using an LC/HDMS^E^ quantitative approach. This shotgun analysis quantified at 1% false discovery rate (FDR) 518 proteins for the SA condition, 530 for the SC condition, 488 for the PC condition, and 377 for the PA condition, with an average of 8 peptides per protein (Supplementary Figures 1, 2). Differential expression was considered only for proteins with a *p*-value ≤0.05 (according to ANOVA) and a fold change of 20%. On this basis, a total of 315 proteins in the PC vs. the SC condition was selected: 155 proteins showed a high level of expression in the PC condition and 160 in the SC condition. For the ATCC group, a total of 403 differentially expressed proteins was selected, 266 of which showed a high level of expression in the PA condition and 137 in the SA condition. The Venn diagram (Figure 2) highlights the shared and the exclusive proteins for each experimental group. The PA and PC conditions shared a considerable amount of proteins (33.4%, *n* = 137), as did the SA and SC conditions (23.4%, *n* = 96).

Comparative analysis of all the significant proteins for each condition failed to reveal a core proteome, which may reflect not only the different physiological states of planktonic and sessile cells but also a relatively small part of the whole bacterial proteome. To obtain a complete description of the functions associated with the differentially expressed proteins for each experimental group, we performed functional analysis using the ClueGO/CluePedia cytoscape plug-in, as described in the Bioinformatics Methods paragraph. Biological process (BP), molecular function (MF), and KEGG ontologies updated to the last version were used for the functional analysis. Two different network specificities (medium and high) were applied to capture different levels of functionality within each ontology for the experimental groups. In addition, a Bonferroni step down correction set to 0.05 was used to keep only the significant processes.

**FIGURE 3:**
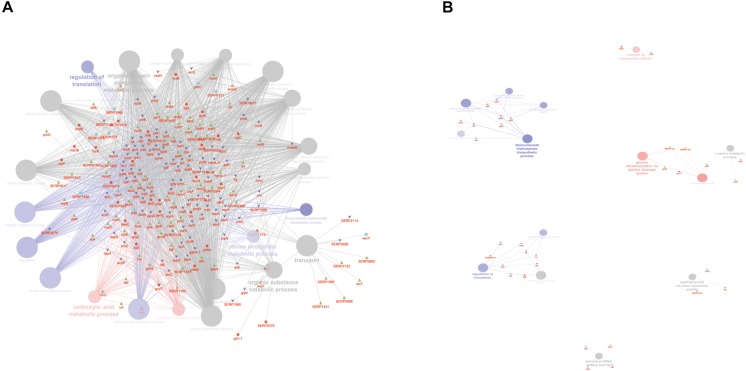
ClueGO cytoscape network of statistically significant proteins. **(A)** GO BP at medium network specificity. Enriched processes in the PA group (red circles), in the SC group (violet circles), and enriched processes common to all conditions (gray circles). Proteins are highlighted in different shapes and colors: PA (red), SA (cyan), PC (green), SC (blue). **(B)** GO BP at high network specificity. Enriched processes in the PA group (red circles), in the SC group (violet circles), and enriched processes common to all conditions (gray circles). Proteins for each condition are highlighted in different shapes and colors: PA (red), SA (cyan), PC (green), SC (blue).

According to the GO BP analysis, differentially expressed proteins at medium network specificity (GO Tree interval 3–8) were organized in 22 GO Terms, 7 of which were highly enriched in the SC group and 2 in the PA group. The other 14 terms were equally enriched in all the groups (Figure 3A). The two processes mainly enriched in the PA group were related to the organic and carboxylic acid metabolic process. In the SC group, the seven processes were related to translation, ribonucleoside triphosphate and amide biosynthetic processes, and ribose phosphate and peptide metabolic processes. Differentially expressed proteins at high network specificity (GO Tree interval 7–15) were organized in 14 GO BP terms, 3 mainly associated with the PA group, 7 with the SC group, and 4 common to all groups. The biological PA processes were related to the removal of superoxide radicals and glycine decarboxylation, and the SC processes were similar to the medium network specificity, except for ATP synthesis coupled to proton transport, ATP synthase activity, and regulation of translation coupled to elongation factor activity (Figure 3B).

**FIGURE 4:**
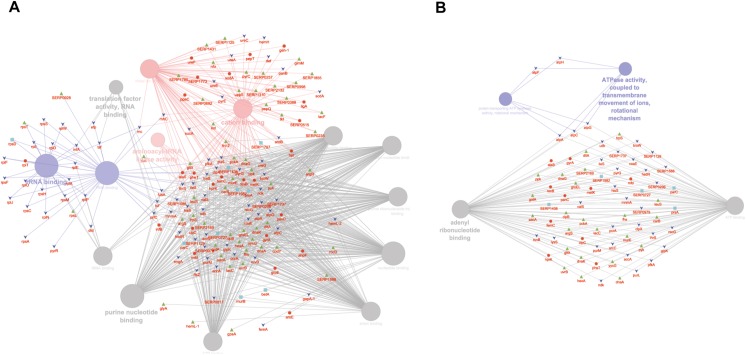
ClueGO cytoscape network of statistically significant proteins. **(A)** GO MF at medium network specificity. Enriched processes in the PA group (red circles), in the SC group (violet circles), and enriched processes common to all conditions (gray circles). Proteins are highlighted in different shapes and colors: PA (red), SA (cyan), PC (green), SC (blue). **(B)** GO MF at high network specificity. Enriched processes in the PA group (red circles), in the SC group (violet circles), and enriched processes common to all conditions (gray circles). Proteins for each condition are highlighted in different shapes and colors: PA (red), SA (cyan), PC (green), SC (blue).

GO MF analysis showed differentially expressed proteins at medium network specificity (GO Tree interval 3–8) organized in 14 GO Terms, two of which were highly enriched in the SC group and three in the PA group. The other nine terms were equally enriched in all the groups (Figure 4A). The three processes mainly enriched in the PA group were related to cation and metal ion binding and tRNA ligase activity. In the SC group, the two processes were related to RNA and rRNA binding (Figure 4A). Differentially expressed proteins at detailed network specificity (GO Tree interval 7–15) were organized in four GO MF terms, two common to all the conditions and two mainly enriched in the SC group. The two SC-enriched MFs were related to proton-transporting ATP synthase, whereas the common MFs were related to ATP and adenyl nucleotide binding and purine ribonucleotide binding (Figure 4B).

**FIGURE 5:**
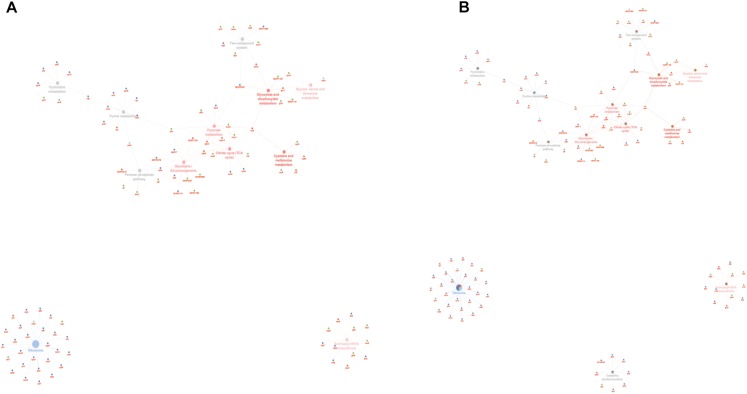
ClueGO cytoscape network of KEGG pathways network from statistically significant proteins. Proteins are highlighted in different shapes and colors: PA (red), SA (cyan), PC (green), SC (blue). Each circle denotes an enriched pathway proportional to the color. **(A)** Enriched processes in the PA group (red circles), in the SC group (violet circles), and enriched processes common to all conditions (gray circles). **(B)** The percentage of the contributing proteins to each pathway is shown in each circle.

To obtain good complementarities to the GO analysis, enrichment analysis was performed for each experimental group against KEGG ontology. Thirteen pathways were globally enriched for all groups: seven were mainly enriched for the PA group and one for the SC group (Figure 5A). The seven pathways mainly enriched in PA were related to several metabolic pathways: glycolysis, pyruvate, TCA cycle, cysteine and methionine, glyoxylate and dicarboxylate, glycine, serine and threonine, and aminoacyl-tRNA biosynthesis. The ribosome was mainly enriched in the SC group. To better assign the remaining pathways to the experimental groups, the percentage of the contributing proteins to each pathway is shown in Figure 5B. Careful analysis of the data associated with Figure 5B showed for the two-component systems (TCSs) an enrichment mainly related to the PA and PC groups, for purine metabolism mainly to SC and SA, pentose phosphate was linked mainly to PC and PA, oxidative phosphorylation to SC followed by SA and PA, and pyrimidine metabolism mainly to SC and PC. GO and KEGG analysis based on ClueGO and Cytoscape correctly retrieved the annotation for two-thirds of all proteins analyzed; the remaining one-third was discussed for the most important functions related to biofilm formation and maintenance.

### Microbial Cytology

**FIGURE 6:**
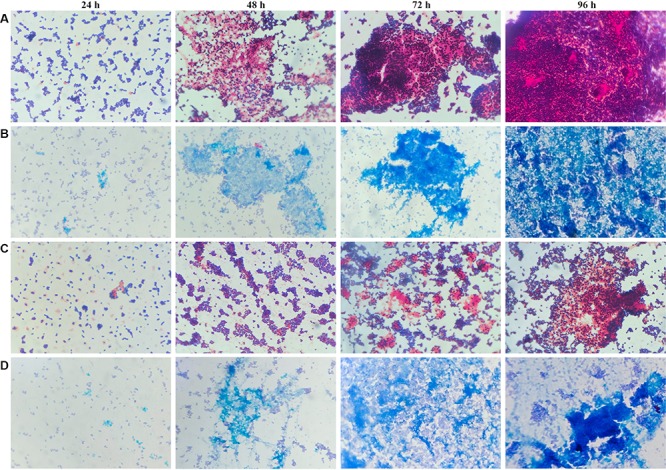
Gram and Alcian blue staining of planktonic *S. epidermidis*. Panel **(A)** shows the planktonic behavior of MRSE ATCC 35984 by Gram staining over time; panel **(B)** depicts the matrix deposited by MRSE ATCC 35984 by means of Alcian blue staining; panel **(C)** illustrates the morphology of MRSE GOI1153754-03-14 stained with Gram, and panel **(D)** shows the EPS matrix deposited by MRSE GOI1153754-03-14 stained with Alcian blue.

To determine whether the planktonic bacteria could aggregate after a long culture period without renewed nutrient supplies, representative images were acquired of Gram and Alcian blue staining of MRSE after 24, 48, 72, and 96 h of planktonic culture (Figure 6). Starting at 48 h, MRSE ATCC 35984 started to aggregate, forming sporadic clusters of bacteria tightly held together by a thin layer of extracellular matrix, as highlighted by the Alcian blue staining. Differently, the clinical isolate (GOI1153754-03-14) had a slower production of extracellular polymeric substances (EPS) compared to the reference strain, which was appreciable starting at 72 h of culture. At the later time points, the clinical isolate demonstrated the ability to not only produce biofilm but also aggregate. Though the two bacterial strains are biofilm-forming, a difference in their behavior after 96 h of culture was evident. MRSE ATCC 35984 formed biofilm-like aggregates at the last experimental time point. The bacterial clumps were characterized by a three-dimensional structure embedded in an EPS matrix.

### CLSM Analysis

**FIGURE 7:**
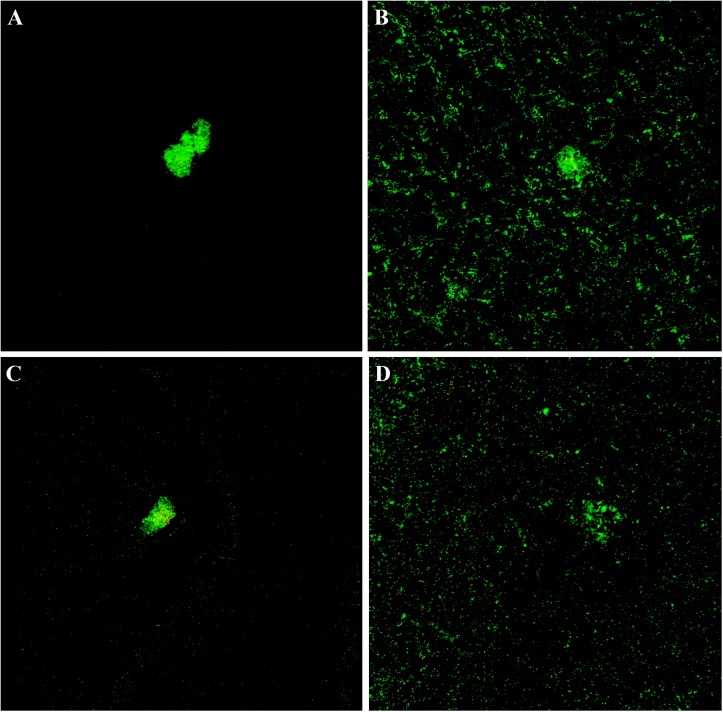
Live and dead staining of planktonic and sessile *S. epidermidis*. Panel **(A)** shows the planktonic and panel **(B)** shows the sessile behavior of MRSE ATCC 35984 after 72-h culture; panel **(C)** depicts the planktonic and panel **(D)** depicts the sessile behavior of MRSE GOI1153754-03-14 at the same experimental time point.

The cytological results were corroborated by CLSM analysis of planktonic and sessile cultures of both MRSE GOI1153754-03-14 and ATCC 35984. Figure 7 presents representative images of the sample. After 72 h of culture, biofilm-like aggregates were clearly visible in both MRSE strains grown in their planktonic form (Figures 7A–C). Quantitative analysis of the live/dead ratio indicated that planktonic culture of the clinical isolates affected bacterial viability. Approximately 18% of the total amount of detected cells resulted dead at the final time point (Table 2). Dead cells can be seen in the core of the bacterial clusters (Figure 7C).

**TABLE 2:** Live/dead cell ratio expressed as percentage.

	**ATCC 35984**	**MRSE GOI1153754-03-14**
Sessile	92.08/7.92	91.65/8.35
Planktonic	97.85/2.15	82.67/17.33

Differently, biofilm-forming MRSE GOI1153754-03-14 and ATCC 35984 showed homogeneous growth on the titanium disks characterized only by the presence of a few bacterial aggregates (Figures 7B–D). The ratio between live and dead bacteria was the same between the clinical isolates and the reference strain (Table 2).

## Discussion

Costerton et al. (1999) defined bacterial biofilm as a “structured community of bacterial cells enclosed in a self-produced polymeric matrix and adherent to an inert or living surface”. The structure of mature biofilms is both complex and well organized; channels provide nutrients to cells that circulate through the biofilm matrix (Donlan, 2002), and bacteria in different regions of the same matrix exhibit different gene expression patterns according to their exposure to external agents (Stewart and Franklin, 2008). This dynamic system is in constant development and it enables bacteria to survive in hostile environments (Costerton et al., 1999). Indeed, biofilm can quietly protect bacteria for long periods, without being detected by the host’s immune system.

Recent efforts to identify biomarkers for biofilm development have profiled the gene expression patterns of sessile bacteria through proteomics to decipher the genetic basis of biofilm formation (Carvalhais et al., 2015a, b; Solis et al., 2016; Freitas et al., 2018). The identification of therapeutic targets or diagnostic biomarkers is crucial to detect latent or chronic infection mediated by low-virulence, biofilm-producing bacteria such as *S. epidermidis*. Hence, investigation of the mechanisms underlying chronic infections would better define markers linked to the presence of a specific bacterium rather than to the host response to an infective status.

With the aim to set a basis for future research, the present study examined the mature biofilms produced by two different *S. epidermidis* strains *via* a proteomic approach to reveal changes in functions related to mature bacterial biofilm compared with the free cell counterpart. While planktonic cells serve as the control to evaluate modulations in the proteomic profile of sessile bacteria, there are important differences in growth phases and developmental stages that need to be kept in mind when reading the following analyses to avert misinterpretation of the data (Azeredo et al., 2016).

### Protein Profile of Sessile Bacteria

We observed overexpressed proteins produced by biofilm-forming *S. epidermidis* related to active metabolic activity (e.g., proteins involved in the synthesis of nucleoside triphosphate and polysaccharides). This phenomenon can be easily explained by the protection that the self-produced EPS matrix confers to sessile bacteria (Fux et al., 2005). After 72 h of static culture on titanium disks, both *S. epidermidis* GOI1153754-03-14 and ATCC 35984 were metabolically active, as assumed from the up-regulation of Ndk. Being involved in the biosynthesis of polysaccharides, this kinase plays an active role in bacterial virulence and adaptation (Yu et al., 2016, 2017). As reported by Yu et al. (2017), Ndk can suppress host defense mechanisms (e.g., phagocytosis, inflammatory response, cell death) or have a cytotoxic effect on host cells, depending on its intracellular or extracellular expression. The main role of Ndk is the biosynthesis of nucleoside triphosphates other than ATP (CTP, UTP, and GTP) (EC 2.7.4.6). By virtue of its housekeeping function, Ndk is a highly conserved enzyme that can be found in both eukaryote and prokaryote cells, where it plays a key role in the synthesis of DNA and RNA (Ray and Mathews, 1992; Lu and Inouye, 1996). Indeed, Ndk was long considered uniquely responsible for nucleoside triphosphate synthesis. This dogma was confuted by Lu and Inouye (1996) who demonstrated that adenylate kinase also possesses Ndk activity through its dual role in the biosynthesis of nucleoside triphosphates and in the synthesis of ADP from AMP with the use of ATP (EC 2.7.4.3). Accordingly, we noted that adenylate kinase was also overexpressed in sessile *S. epidermidis* GOI1153754-03-14.

Similarly, 3-oxoacyl-[acyl-carrier-protein] reductase FabG (EC 1.1.1.100) was overexpressed in sessile MRSE. A member of the ketoacyl reductase family, FabG plays a crucial role in the elongation cycles required to synthesize long-chain fatty acids in the type II fatty acid biosynthesis (FAS II) process (Lai and Cronan, 2004). It is also involved in the phospholipidic membrane adaptation of bacteria growing in a sessile state. It has been suggested that reduced membrane fluidity enhances survival in a harsh environment probably because there are fewer exchanges between the protected bacteria and their surroundings (Dubois-Brissonnet et al., 2016). Furthermore, its ubiquitous presence and essential biological role make FabG a possible target for the development of a broad-spectrum antibiotic (Heath and Rock, 2004). Brinster et al. (2009) demonstrated, however, that major Gram-positive pathogens might not require the FAS II process for their survival since they can assimilate fatty acids straight from host serum.

### Protein Profile of Planktonic Bacteria

Based on the proteins we identified, it appears that many of the changes in the proteomic profile of both *S. epidermidis* strains occurred when planktonically cultured. Our data revealed the overexpression of proteins linked to bacterial stress and to anaerobic growth typical of sessile culture conditions. When embedded in a mature biofilm matrix, bacteria must deal with conditions of scarce oxygen availability, metabolic waste, and high cell density (Fey and Olson, 2010). These environmental factors have a crucial role in biofilm development because they can trigger stress response genes and shift staphylococcal metabolism toward anaerobiosis to compensate for the oxygen shortage, as demonstrated by Rani et al. (2007).

Specifically, after 72 h of culture, the putative universal stress protein was significantly up-regulated in both *S. epidermidis* strains growing in planktonic form. The universal stress protein A (uspA) superfamily is a conserved group of proteins expressed in a variety of species including bacteria, fungi, Archaea, and insects (Kvint et al., 2003). A high cell density in a closed environment without renewed nutrient supplies inevitably alters physiological cell balance; harsh conditions (i.e., nutrient deprivation, decreased pH, and exposure to oxygen and nitrogen species) predictably lead to global stress responses (Foster, 2007). Similarly, organic hydroperoxide resistance protein-like 1, another cytoplasmic protein expressed in response to oxidative stress, was up-regulated in both *S. epidermidis* strains grown in planktonic aggregates. This protein belongs to the peroxiredoxin family, which is considered the primary cellular protector system against oxidative stress in all living organisms; it contributes to detoxifying organic peroxides and favoring microbial survival (Cao and Lindsay, 2017).

Staphylococci have evolved many defense strategies to survive in the presence of exogenous and endogenous oxidants (Gaupp et al., 2012). Furthermore, as cell density increases, the quorum sensing (QS) system is activated to coordinate the expression of different genes through small signaling molecules called autoinducers (Xu et al., 2006). LuxS is involved in the synthesis of the autoinducer-2, a QS signaling pheromone expressed by both Gram-positive and -negative bacteria (EC 4.4.1.21) (Piras et al., 2012; Kırmusaoğlu, 2016), and its up-regulation is known to be closely connected to QS stress (Li et al., 2008; Arciola et al., 2012). Conversely, LuxS enzyme inhibitors have been demonstrated to actively increase the virulence of *S. epidermidis*, boosting its ability to form biofilm (Xu et al., 2006). Once again, the overexpression of this protein in planktonic aggregates of *S. epidermidis* suggests the activation of a protective mechanism against the harsh environmental condition after 72-h culture.

The ability to adapt in response to stressful situations is crucial for bacterial survival and *S. epidermidis* is an extremely versatile microorganism. Because it is a facultative anaerobe, it can cope with oxygen shortage and survive in a wide range of oxygen concentrations by switching between aerobic and anaerobic pathways (Uribe-Alvarez et al., 2016). Though planktonic aggregates were cultured under aerobic conditions, the clinical isolates also expressed two enzymes related to oxygen shortage: alcohol dehydrogenase (EC 1.1.1.1) and L-lactate dehydrogenase (EC 1.1.1.27). In anaerobic growth in the absence of electron acceptors, staphylococci are able to metabolize glucose to pyruvate, and then to reduce pyruvate to lactate, ethanol, and acetate in a process of mixed-acid fermentation (Shan et al., 2012). Fuchs et al. (2007) reported that the expression of lactate dehydrogenase and alcohol dehydrogenase is highly induced in *S. aureus* when the electron transport chain is interrupted, indicating that oxygen concentration alone might not be sufficient to regulate the genes involved in this process. They went on to speculate that fermentation in *S. aureus* might be activated also by the changes in membrane potential or in the levels of NADH and/or state of components of the respiratory chain (Fuchs et al., 2007).

In our proteomic analysis, overexpression of ArsC 1 was observed in the sessile form and that of ArsC 2 was observed in the planktonic aggregates of *S. epidermidis* clinical isolates. Arsenate reductase is a complex system comprising at least three enzymes that reduce As (V) in As (III) (Zegers et al., 2001). Not only is ArsC able to reduce arsenic but it can act as a phosphatase in specific conditions. Sequence analysis using the Pfam database^3^ highlighted the presence of an LMW PTPases domain in both enzymes. Moreover, the phosphatase active site cys10, which catalyzes the dephosphorylation reaction, is extremely sensitive to oxidation (Messens et al., 2003) that impairs this function. The importance of this phosphatase activity in biofilm maintenance and release was demonstrated in *P. aeruginosa* where increased expression of phosphatase TbpA led to a signal cascade and the detachment of the mature biofilm (Ueda and Wood, 2009). Taken together, these findings corroborate our observations. Since ArsC1 was increased in the sessile form, the anaerobic environment associated with mature biofilm could reactivate the phosphatase activity and lead to the detachment of mature biofilm. This phenomenon is not possible in planktonic aggregates, however, where ArsC2 and the LMW PTPases domain are subjected to a higher oxygen concentration than the sessile form.

The expression of enzymes related to anaerobic growth does not exclude the possibility that other proteins may be identified, such as isocitrate dehydrogenases (EC 1.1.1.42) related to the tricarboxylic acid (TCA) cycle. The physiological heterogeneity of bacterial populations enables the expression of distinct metabolic pathways related to specific biological activities depending on the gradients of metabolic substrates and products present in the local environment, particularly when embedded in biofilm (Stewart and Franklin, 2008). Accordingly, our analysis of proteins expressed by planktonic MRSE aggregates revealed phenomena linked to bacterial stress and growth under anaerobic conditions and a biofilm-like behavior of planktonic cells.

We performed cytological and CLSM analyses to verify the hypothesis that bacteria can aggregate and secrete EPS as a survival mechanism. The preliminary evidence strengthened our hypothesis that 72-h culture under vigorous agitation can create a stressful growing environment that triggers the aggregation of microorganisms in a biofilm-like matrix as a means to survive harsh environmental conditions. The aggregation of free-floating staphylococci to survive unfavorable culture conditions was previously reported by Haaber et al. (2012). In particular, they concluded that bacterial aggregates display a higher metabolic activity compared to planktonic or cells embedded in biofilm. These findings could explain those reported in the present study, suggesting that high metabolic activity of aggregates could lead faster to a nutrient- and oxygen-deprived environment, and subsequently, to stress response and anaerobiosis, whereas a mature biofilm seems to handle more efficiently adverse conditions (Haaber et al., 2012).

### Adhesion Proteins Showed Different Expression Dynamics in Planktonic vs. Sessile Bacteria

Bifunctional autolysin Atl was found overexpressed in the SC group (Supplementary File 1). This surface-associated proteinaceous adhesin is known to be involved in cell wall turnover, cell division, and cell lysis (Paharik and Horswill, 2016). As described elsewhere, the expression of this adhesion protein was decreased during the first 12 h of biofilm growth compared to the planktonic bacteria but rose 10-fold after 48 h, suggesting an important role later in the biofilm cycle (Rohde et al., 2010). In the SC group, this overexpression was detected at 72 h when autolysis was probably massively induced and eDNA released. In contrast, this protein was massively expressed in the PA and not in the SA group, partially supporting a biofilm-like behavior at least for the planktonic ATCC.

Careful analysis of the dataset revealed an alternative protein, *N*-acetylmuramoyl-L-alanine amidase Sle1, through which only the SA group controlled its adhesive activity in the sessile form. This protein is a 35-kDa surface-associated protein involved in cell wall metabolism and in some adhesion processes; it binds to fibrinogen, fibronectin, and vitronectin (Heilmann et al., 2003). Based on these data, different mechanisms by which sessile cells control biofilm formation and management can be imagined, though further analyses are needed to clarify the role of Atl overexpression in planktonic ATCC cells.

### Several TCSs Are Differentially Modulated in Planktonic and Sessile Bacteria

As in other pathogenic bacteria, TCSs regulate bacterial metabolism, development, survival, and virulence in addition to the important role they play in *S. epidermidis* biofilm formation. KEGG analysis highlighted several proteins associated with the TCS as being enriched in the two planktonic conditions (PA and PC). Several systems and proteins were specifically detected. As depicted in Figure 5B, at least 11 proteins were mapped to the TCSs pathway. One of the most represented was the essential YycFG (or WalKR) TCS detected by KEGG analysis (Figure 5B). This system was mainly overexpressed in both PA and PC in which, after 72 h of culture, CLSM analysis confirmed biofilm-like aggregates. Corroborating our experimental data, a recent study suggested that YycF (WalR) up-regulates cell aggregation and other biofilm-related functions (Xu et al., 2017).

Also, we identified in our data the icaB protein, encoded by the icaADBC operon involved in deacetylation and activation of PIA. This protein was overexpressed in the SA and the PC group compared to their counterparts. Our dataset did not detect the biofilm PIA synthesis protein icaA in the main regulated protein of the YycFG system (Xu et al., 2017), probably due to the late sampling time (72 h). Nonetheless, it is intriguing to note that there was a direct relation between overexpression of the YycFG system and icaB overexpression only for the clinical isolates, hinting at a possible role of this system in the biofilm-like behavior of the PC group.

A previous study linked YycFG system expression to altered fatty acid biosynthesis and bacterial membrane composition (Mohedano et al., 2005). In this view, over-representation of the response regulator protein VraR (vraR gene, Q8CNP9) observed in the planktonic aggregates might suggest a restructuring of the bacterial cell wall, resembling, once again, the biofilm-like behavior of the sessile bacteria. Besides conferring resistance against antibiotics acting on the cell wall (e.g., vancomycin) (Qureshi et al., 2014), VarR is also a member of the TCS VraS/VraR involved in the positive regulation of peptidoglycan biosynthesis (Kuroda et al., 2003). In addition, the D-alanine-D-alanyl carrier protein ligase (*dlta* gene, Q8CT93) plays an important role in modulating the cell wall properties in Gram-positive bacteria. A recent study performed on Gram-positive bacterium *Parvimonas micra* linked this protein to both bacterial growth and biofilm production (Liu and Hou, 2018). Hence, the higher expression of this protein in the free-floating bacteria is consistent with and reinforces the suggested biofilm-like behavior of planktonic bacteria.

Despite the enrichment obtained with ClueGO, the lack of a complete annotation for *S. epidermidis* ATCC 35984 (RP62A) led to undersampling in the TCS analysis. To overcome this problem, we performed a manual survey of data and discovered another important *S. epidermidis* TCS: the SaeRS TCS was detected in a supervised manner and our data showed a relevant overexpression only in the SA group. An equal amount of this system was detected in both biofilm and planktonic aggregates of the clinical strain (Supplementary File 1). As previously reported, deletion of SaeRS altered bacterial autolysis, increased eDNA release, and decreased bacterial cell viability in both the planktonic and the biofilm state (Lou et al., 2011). These data may support the increase in the biofilm-forming capacity of the SC group, together with the overexpression of the autolysin Atl. Moreover, expression of this system in the PC group at levels comparable to SC could support a biofilm-like behavior, as confirmed from the microbial cytology and the CLSM. Such a similar medium-low expression level of SaeRS in the PC compared to the ATCC may influence strain viability, as confirmed here by the CLSM analysis and previously (Lou et al., 2011).

### Planktonic Bacteria Are Strongly Involved in Central Metabolism

Other KEGG metabolic pathways overrepresented in planktonic aggregates include the so-called central metabolism (i.e., glycolysis/gluconeogenesis, pyruvate metabolism, and TCA cycle). We identified the pyruvate dehydrogenase complex (*pdhA* gene, Q8CPN3; *pdhB* gene Q8CPN2), probable malate:quinone oxidoreductase-1 (*mqo-1* gene, Q8CN91), probable malate:quinone oxidoreductase-3 (*mqo-3* gene, Q8CN91), and fumarate hydratase class II (*fumC* gene, Q8CNR1). Altogether, these proteins indicate active bacterial metabolism and an overall trend toward carbohydrate degradation. Moreover, the overexpression of putative aldehyde dehydrogenase SERP1729 (*SERP1729* gene, Q5HMA0), alcohol dehydrogenase (*SERP0257* gene, Q8CQ56), and zinc-type alcohol dehydrogenase-like protein SERP1785 (*SERP1785* gene, Q5HM44) strongly support the previous finding of anaerobic bacterial growth, besides the initial aerobic culture condition. This reflects a further attempt of the planktonic bacteria to resemble the sessile biofilm-forming community.

### Sessile Bacteria Are Mainly Involved in Ribosome Pathway, Purine, and Pyrimidine Biosynthesis

Although planktonic aggregates showed most of the metabolic changes, the bacteria grown under the sessile condition were more active in ribosome, purine, and pyrimidine metabolism (Figure 5B). The overexpression of the ribosome pathway proteins indicates overall involvement of the sessile strains in active metabolism, featured by a steady turnover of the translational apparatus and the production of accessory macromolecules required for accurate throughput protein biosynthesis. Consistent with previous evidence, the biofilm-producing phenotypes (i.e., sessile bacteria) expressed a high abundance of Ndk protein, along with other proteins belonging to purine and pyrimidine metabolism. Recent investigations have demonstrated the importance of *de novo* purine biosynthesis for biofilm formation (Haas and Défago, 2005; Ge et al., 2008; Ruisheng and Grewal, 2011; Kim et al., 2014). Using *Pseudomonas fluorescens* as a biofilm-producing model, Yoshioka recently applied transposon-mediated mutagenesis of different purine biosynthesis genes to obtain purine auxotrophic bacteria with a significantly reduced biofilm formation capability (Yoshioka and Newell, 2016).

In our study, the activation of *de novo* biosynthesis of purine was also confirmed by the overexpression of the *pur L* and *pur M* genes, encoding, respectively, for phosphoribosylformylglycinamidine synthase and phosphoribosylformylglycinamidine cyclo-ligase. Both enzymes are sequentially involved in the *de novo* biosynthetic pathway of inosine monophosphate, a purine precursor. Phosphoribosylformylglycinamidine synthase catalyzes the ATP-dependent conversion of formylglycinamide ribonucleotide (FGAR) and glutamine to yield formylglycinamidine ribonucleotide (FGAM) and glutamate. In turn, phosphoribosyl formylglycinamidine cyclo-ligase converts FGAM into aminoimidazole ribonucleotide (AIR), ADP, and inorganic phosphate in an ATP-dependent manner (Li et al., 1999). Nevertheless, overexpression of other genes such as *xpt*, *ure A*, *ure C*, and *arc C* suggest the simultaneous activation of the salvage pathway for purine biosynthesis, resulting in enhanced production of purine, most likely required for biofilm production and maintenance.

Pyrimidine biosynthesis was also found to play a crucial role in the biofilm production phenotype (Garavaglia et al., 2012; Ahmar et al., 2019). In the present study, *de novo* biosynthesis of pyrimidine is supported by the identification, among others, of orotate phosphoribosyltransferase (*pyr E* gene, Q8CSW7). This protein is involved in the first step of uracyl monophosphate (UMP) biosynthesis by catalyzing the transfer of a ribosyl phosphate group from 5-phosphoribose 1-diphosphate to orotate, leading to the formation of orotidine monophosphate (OMP) (Henriksen et al., 1996). Also, the overexpression of CTP synthase (citidine triphosphate synthase, *pyr G* gene, Q8CNI2) supports the *de novo* biosynthesis of the nucleotide cytosine by catalyzing the ATP-dependent amination of the UTP pyrimidine ring at 4-position to obtain CTP using either L-glutamine or ammonia as a nitrogen source (Endrizzi et al., 2004). Other proteins such as uridine kinase (udk gene, Q8CSB2) highlight the effort bacteria mount in pyrimidine biosynthesis by activating the salvage pathway, resulting in pyrimidine biosynthesis in a more cost-effective way (Beck and O’Donovan, 2008).

## Conclusion

The biofilm-like phenotypes of floating bacteria are an emerging concept. Recent evidence of biofilm-like aggregates of staphylococci in synovial fluids has been described (Dastgheyb et al., 2015; Perez and Patel, 2015). The dogma of biofilm formation following bacterial adhesion to a biotic or abiotic surface is slowly changing. Currently, it is unclear whether the expression of biofilm-related genes is triggered by attachment or is consequent to altered nutrient and oxygen of supply, metabolic product accumulation, and/or consequent to activation of a QS mechanism (Becker et al., 2001). Even though the majority of studies aim to elucidate the phases of biofilm formation starting from the bacterial adhesion to a surface, there are more and more articles in the literature reporting the aggregation of free-floating bacteria embedded in an extracellular matrix (Alhede et al., 2011; Haaber et al., 2012; Crosby et al., 2016; Kragh et al., 2016). In our study, the choice of the unique late time point revealed an important clue for future investigation into the biofilm-like behavior of planktonic cells in harsh culture conditions. Though preliminary, the present results may contribute to changing the perspective on comparative proteomic strategies in the study of mature bacterial biofilm and challenge the dogma of biofilm formation on surfaces.

## Author Contributions

MB, LB, PR, and AL conceived and designed the study. MB and AB collected the samples and performed the cytological analysis. AS, CP, LP, and VG performed the proteomic and statistical analysis. MB wrote the first draft of the manuscript. AL, PR, LP, and AS wrote sections of the manuscript. MB, AS, LB, PR, and AL revised the manuscript critically. All authors contributed to manuscript revision, read and approved the submitted version.

## Conflict of Interest Statement

The authors declare that the research was conducted in the absence of any commercial or financial relationships that could be construed as a potential conflict of interest.

## Footnotes

1 www.matrixscience.com

2 http://bioinfogp.cnb.csic.es/tools/venny/

3 https://pfam.xfam.org/
