# Supplementary material for: Proteomic Analysis Reveals a Biofilm-Like Behavior of Planktonic Aggregates of Staphylococcus epidermidis Grown Under Environmental Pressure/Stress
Source: Front Microbiol. 2019 Sep 6;10:1909. doi: 10.3389/fmicb.2019.01909 (PMC6743020; doi:10.3389/fmicb.2019.01909)
Supplement: Supplementary file 1 [file Table_1.DOCX]

Supplementary Material

# Supplementary Data

A list of all the proteins identified and quantified by the label-free shotgun approach is reported in Supplementary File 1.

## Supplementary Figures

**
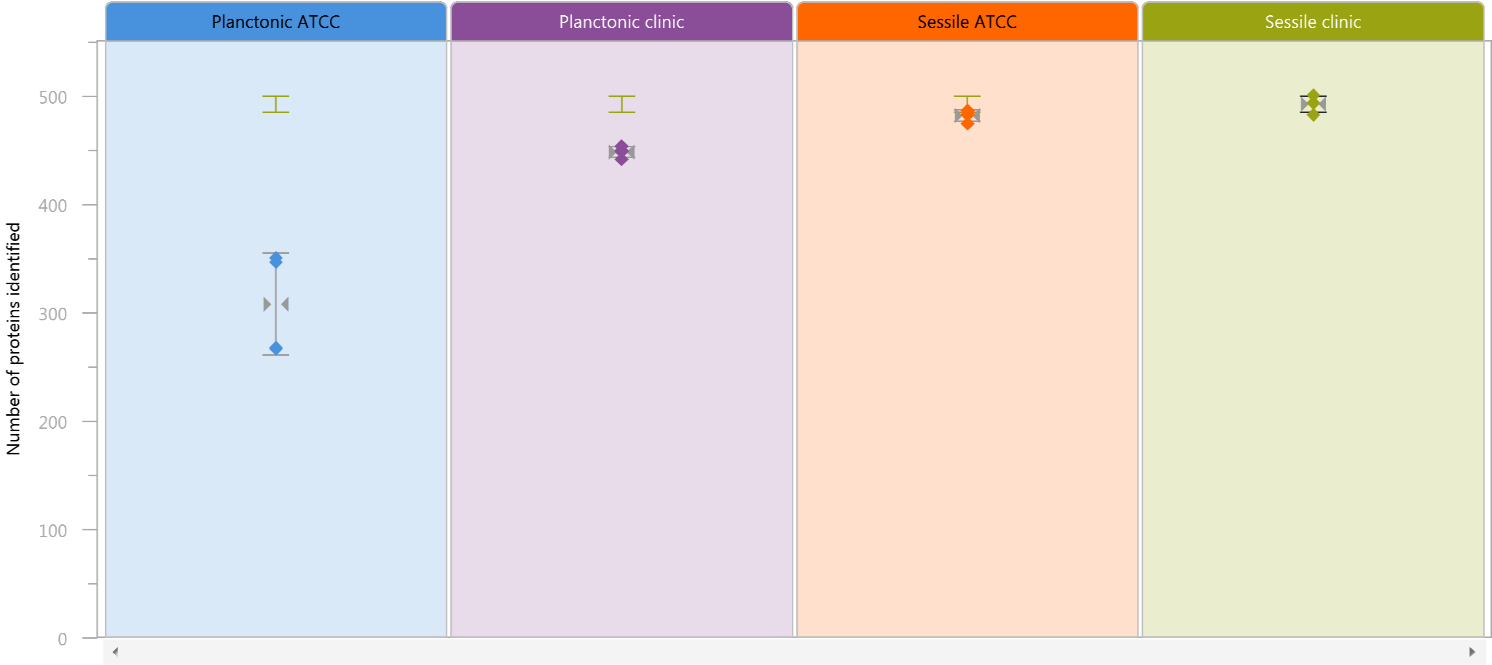
**

**Supplementary Figure 1.** Proteins identified and quantified by the label-free shotgun approach in each experimental condition


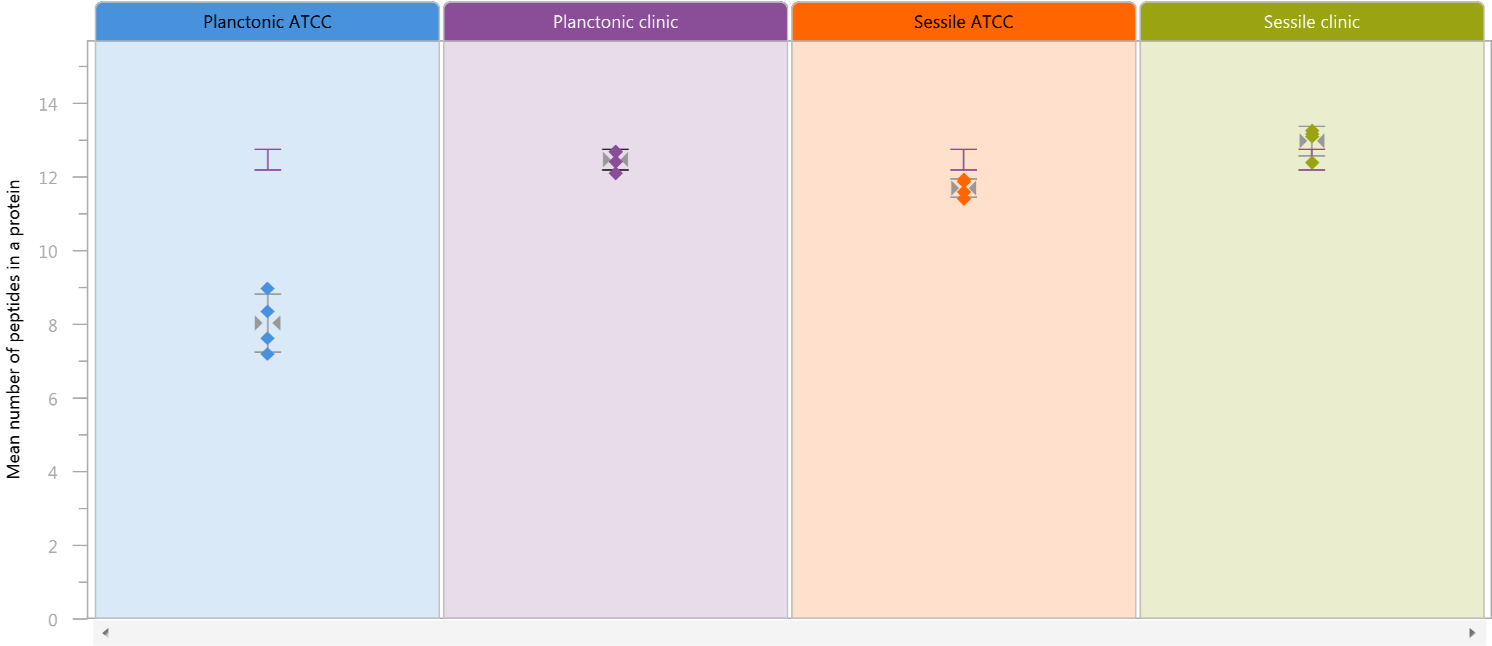


**Supplementary Figure 2.** Peptides identified and quantified by the label-free shotgun approach in each experimental condition.
